# Outcomes and Complications in a Case Series of 39 Total Laparoscopic Prophylactic Gastropexies Using a Modified Technique

**DOI:** 10.3390/ani11020255

**Published:** 2021-01-20

**Authors:** Veronica Giaconella, Riccardo Grillo, Roberto Giaconella, Roberto Properzi, Rodolfo Gialletti

**Affiliations:** 1 Clinica Veterinaria Giaconella, via Eugenio Checchi 57, 00157 Roma (RM), Italy; riccardogrillo3@gmail.com (R.G.); rgiaconella@gmail.com (R.G.); 2 Studio Veterinario Properzi, via Santa Maria del Campo 16, 16035 Rapallo (GE), Italy; dr.properzi@libero.it; 3 Department of Veterinary Sciences, University of Perugia, via S. Costanzo 4, 06126 Perugia (PG), Italy

**Keywords:** GDV, prophylactic gastropexy, total laparoscopic gastropexy, barbed suture

## Abstract

**Simple Summary:**

Gastric dilatation and volvulus is a very severe condition that is most commonly seen in large and giant deep-chested dogs, although any dog may be affected. Recently, an increasing number of breeders and owners have become aware of the benefits of prophylactic gastropexy. Many techniques have been developed to perform gastropexy, but laparoscopic surgery, having very low levels of morbidity and invasiveness, fits well with the concept of prevention. The aim of this study is to validate a rapid, modified total laparoscopic prophylactic gastropexy technique with a low rate of complications. The results show that this procedure is safe and effective. Using this technique, it is possible to respect animal welfare and prevent the development of a life-threatening syndrome.

**Abstract:**

Laparoscopic-assisted, laparoscopic, and endoscopic gastropexy techniques have been proven successful in recent years. Thanks to minimal invasiveness, low morbidity, and fast recovery, total laparoscopic gastropexy techniques have been gaining popularity. The objective of this study was to describe the use of a modified minimally invasive technique to perform prophylactic gastropexy in dogs. A case series study of 39 client-owned dogs was undertaken from June 2019 to August 2020. Each dog underwent total laparoscopic prophylactic gastropexy using a simple continuous barbed suture line and two laparoscopic needle holders without incising the seromuscular layer of the stomach and the abdominal wall. Surgical time, the number of stitches, and the length of suture were recorded. Telephone checks, owner questionnaires, and ultrasonographic exams were used to evaluate the effectiveness of the procedure after surgery. The median gastropexy surgical time was 12 min (range 4–30 min), and the median length of the suture line was 3 cm (range 2–4 cm). The last follow-up check was carried out 9 months (mean, range 3–14 months) after surgery, and all ultrasonographic exams (n = 29) showed an intact gastropexy. Intraoperative and postoperative complications were noted. This total laparoscopic gastropexy technique was found to be safe, fast, simple, and with a low morbidity rate. It appears to be a new alternative to other methods of prophylactic gastropexy; however, further research in this area is warranted.

## 1. Introduction

Gastropexy is a surgical procedure in which the stomach is permanently attached to the right internal abdominal wall. In 1979, it was used for the first time to treat and prevent the recurrence of gastric dilatation-volvulus (GDV) [1]. Canine GDV syndrome is an acute and potentially life-threatening condition that mainly affects medium to large and giant-breed dogs. A wide range of pathogenetic mechanisms have been hypothesized, but the exact etiology is not yet known [2,3]. Gastropexy has been proposed to prevent disease recurrence in dogs that have undergone surgery because of GDV and is used as a prophylactic surgery in predisposed dogs. Effective gastropexy treatment has been shown to decrease the recurrence rate of gastric torsion from 80% to less than 5% [1,2,3,4,5,6,7,8,9,10,11,12,13]. Nowadays, prophylactic gastropexy can be performed by laparotomy, laparoscopic- or endoscopic-assisted techniques, or full laparoscopy [1,2,3,5,7,8,9,10,11,12,14,15,16,17,18]. Thanks to their minimal invasiveness, low morbidity, and fast recovery rates, total laparoscopic gastropexy techniques have gained popularity. The most demanding and time-consuming aspect of these techniques is knot tying, but barbed suture and specific laparoscopic suture devices can now be used to overcome this difficulty [3,7,8,9,12,19,20,21,22,23,24]. Usually, a welded loop at the end of the barbed suture can anchor the suture line before the simple continuous pattern is continued. Instead of a loop, another type of barbed suture provides a button as a self-anchoring system. Until now, an incision or a partial thickness peritoneal abrasion has usually been performed before suturing to stimulate a tissue reaction that leads to the creation of permanent adhesion between the gastric seromuscular layer and the right transversus abdominis muscle. However, little is known about the need for this step, although it is likely to increase gastropexy surgical time. It has already been demonstrated that a knotless unidirectional barbed suture can form a pexy without creating intentional trauma to the gastric area or the abdominal wall [23].

The objectives of this study were to: (1) Verify the formation of a lasting gastropexy without performing an incision or abrasion on the seromuscular layer of the stomach and the abdominal wall using a knotless unidirectional barbed suture with a self-anchoring button and two laparoscopic needle holders; (2) To report on the short- and long-term intraoperative outcomes and complications in client-owned dogs undergoing a total laparoscopic prophylactic gastropexy. We hypothesized that this technique would be safe, fast, and would create a lasting gastropexy.

## 2. Materials and Methods

This prospective study was a clinical trial in which a total laparoscopic prophylactic gastropexy was performed on client-owned dogs between June 2019 and August 2020 using a simple continuous barbed suture line and two laparoscopic needle holders, without incising the seromuscular layer of the stomach and the abdominal wall. Each owner signed an informed consent form in which the purpose and the protocol of the study and potential risks, including the risk of gastropexy failure and risks usually associated with anesthesia, were described. The surgery time (from first skin incision to last port closure), gastropexy time (from the insertion of the instruments in the ports to suture fulfillment), length of suture, number of stitches, performance of other laparoscopic or extra-abdominal procedures, and intraoperative complications were noted. The length of hospitalization, presence of postoperative complications, date of ultrasonography exams, clinical follow-up examinations, questionnaires, and telephone checks were recorded. The inclusion criteria were that dogs undergoing total laparoscopic prophylactic gastropexy procedures were healthy and had a minimum follow-up period of 3 months. Exclusion criteria were conditions that could have increased the risk of adverse effects from anesthesia, such as metabolic or systemic diseases, and preoperative physical examination or blood work abnormalities.

Preoperatively, the complete blood count, serum biochemistry profiles, and a preanesthetic examination were performed on each dog to assess its general health status. Dogs were fasted for 12 h prior to surgery. Anesthetic and analgesic management protocols were selected by the attending anesthesiologist at the time of surgery. General anesthesia was induced with propofol approximately 20 min after premedication and maintained with isoflurane delivered in 100% oxygen via a rebreathing circuit. All dogs received 0.2 mg/kg meloxicam subcutaneously immediately before induction [24,25]. Intraoperative analgesia was obtained by an IV loading dose of 0.2 µg/kg sufentanil followed by a 0.5 µg/kg/h rate infusion, which was maintained until the end of the surgery. Intraoperative monitoring was performed using a multiparametric monitor (heart and respiratory rates, electrocardiography, arterial blood pressure, pulse oximetry, end-tidal carbon dioxide tension (EtCO_2_), capnography). A mechanical ventilator was used to maintain the EtCO_2_ in a range of 30–45 mmHg. Ringer’s lactate solution was administered at a constant rate of 10 mL/kg/h during surgery.

**Figure 1:**
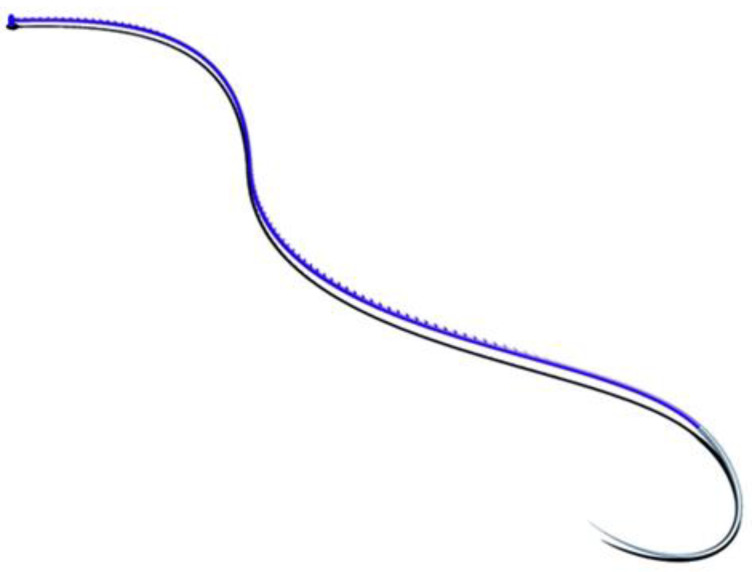
Knotless, synthetic, monofilament, absorbable barbed suture with a button final lock system (Filbloc, polydioxanone (PDO) monofilament long term absorption, Assut Europe, Roma, Italy).

**Figure 2:**
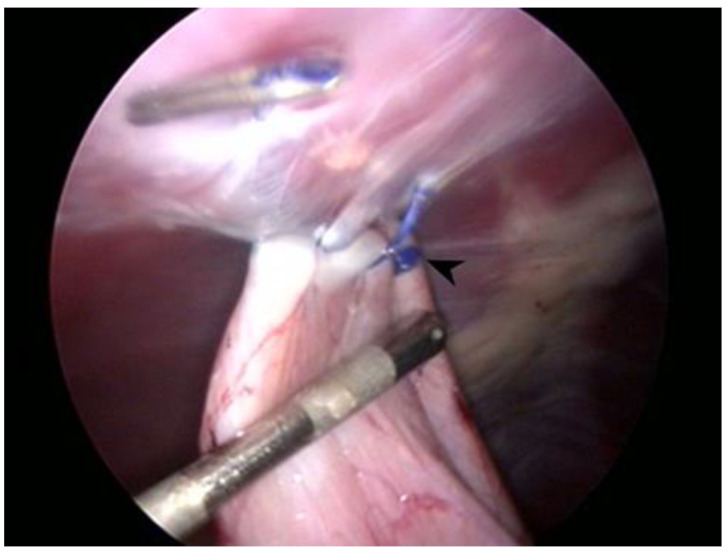
Laparoscopic image at the end of the gastropexy procedure while measuring the suture length. Note the button at the beginning of the suture (arrowhead) and the endoclip at the opposite end.

All dogs were prepared for aseptic surgery. The ventrolateral abdomen was clipped and scrubbed down as in a traditional laparotomy procedure. The surgeries were performed by two surgeons with more than 15 years of experience with laparoscopic procedures and over 3 years of experience with performing total laparoscopic prophylactic gastropexy procedures. Each dog was placed in dorsal recumbency. In all procedures, a three-port technique was used, as previously reported [23,24,25]. The first port (T1) was created via the open Hasson approach. Specifically, a 12 mm cannula (T1) (Trocar Hybrid, X-ONE, MediLine, S. Gregorio, Italy) 1 cm caudal to the umbilicus was placed in the right paramedian position to avoid overlap with the falciform ligament. Capnoperitoneum was established by a CO_2_ automatic insufflator at a pressure level of 10–12 mmHg. A 5 mm 0° telescope (HOPKINS II, KARL STORZ Endoscopy, Goleta, CA, USA) was inserted through T1. Via direct observation, the second (T2) and third ports (T3) were placed approximately 8–10 cm apart cranially and caudally to T1 using two 6 mm EndoTIPs (TERNAMIAN EndoTIP Cannula, KARL STORZ Endoscopy, Goleta, CA, USA) in the right paramedian position. Once all ports had been established, the insufflator pressure was reduced to 8 mmHg. A telescope was inserted in T2, and two laparoscopic needle holders (LOFT-LINE needle holder, Tontarra Medizintechnik GmbH, Wurmlingen, Germany) were introduced in T1 and T3. After abdominal exploration, a self-retaining knotless unidirectional barbed suture with a polydioxanone (PDO) button (0 Filbloc, 15 cm, PDO monofilament long term absorption, Assut Europe, Roma, Italy; HSRG 36 mm-1/2 tapper cutting, Assut Europe, Roma, Italy) (Figure 1) was held by a needle holder and brought into the abdomen through T1. The needle was then held intracorporeally with the other needle holder and positioned as previously described [22]. The first bite was taken on the seromuscular layer of the pyloric antrum, halfway between the lesser and greater curvatures, and the stomach was anchored at the abdominal wall in a right paracostal position, 2–3 cm caudal to the last rib. Before proceeding with the simple continuous suture pattern, it was necessary to apply tension to the suture line to prevent backward movement and to allow adequate contact between the stomach and the transversus abdominis muscle. Two to three more bites were performed 5–7 mm apart, and then a titanium endoclip (Endo Clip 5 mm, Medtronic, Minneapolis, MN, USA) was applied on the suture line to create a landmark for the short- and long-term follow-up examinations and the suture was cut intracorporeally (CLICKline Scissors, KARL STORZ Endoscopy, Goleta, CA, USA). Once the procedure was completed (Figure 2), the strength of the gastropexy line and the length of the suture were both evaluated with a 5 mm probe (Palpation Probe with cm marking, KARL STORZ Endoscopy, Goleta, CA, USA). After abdomen deflation, port site closure was performed in a standard manner using 0 or 2-0 polyglyconate to oppose the muscle layer and the subcutaneous layer, and skin closure was achieved using tissue adhesive (3M Vetbond, 3M Health Care, St. Paul, MN, USA). If other elective procedures (i.e., ovariectomy or castration) had to be performed, they were done after gastropexy. The total surgery time (from first skin incision to last port closure), gastropexy time (from the insertion of the instruments in the ports to suture completion), length of suture, number of bites, performance of other procedures, and presence of intraoperative complications were recorded.

All dogs were discharged on the day of surgery. Recommended postoperative care included treatment with meloxicam (0.1 mg/kg orally once a day) for 5 days, activity restriction to kennel-rest or walking on a short leash, and subdivision of the daily food ration into 5–6 small meals per day for 1 week.

The owners were contacted 1 week after surgery to collect information about the general health of the dog and the surgical site condition. A 2-week postoperative clinical follow-up examination was recommended for all dogs. Telephone checks and questionnaires were carried out by a single investigator, and the minimum follow-up period required for the questionnaire was 3 months. Telephone interviews were repeated at 6 and 12 months after surgery. Clinical variables noted at each follow-up examination were the presence of lethargy, pain, or abdominal discomfort on manipulation; episodes of regurgitation or vomiting; inappetence; weight loss; signs of gastric dilation; surgical site complications; behavioral changes; and changes in quality of life. To standardize the procedure, all questions were asked to the owners in the same order, and the answers could only be yes or no. The question about patient quality of life concerned each dog’s attitude, mood, and willingness to participate in play or interact. This variable was rated on a scale from 1 (poor) to 10 (excellent). Further information was requested only in cases of affirmative answers.

The minimal required follow-up time for an ultrasonographic exam was 3 months, and a 6-month examination was advised for all owners. Each examination was performed by one of two radiologists with over 20 years of experience. Dates of examination and findings were recorded. Dogs were fasted for 12 h prior to ultrasound imaging. The region in which the gastropexy should have been detected was clipped, and focal ultrasounds were performed with the dog in a standing position. The gastropexy site was confirmed when the pyloric antrum in contact with the peritoneal surface of the abdominal wall was identified and the absence of the slide sign was observed [23].

Descriptive statistical analyses were performed using statistical software (XLSTAT-Biomed statistical software; Addinsoft, New York, NY, USA). Continuous numerical variables were not normally distributed based on the rejection of the null hypothesis using the Shapiro–Wilk test. Numeric variables were reported as the median and range, while categorical variables were reported as ratios and percentages.

## 3. Results

A total of 57 dogs underwent total laparoscopic prophylactic gastropexy from June 2019 to August 2020. Of the 57 cases, 10 never returned for any form of follow-up examination, and 8 did not reach the minimum required follow-up time. The inclusion criteria requirements were met by 39 client-owned dogs, of which 11 were male and 28 were female (23 intact, 5 spayed). The median age at the time of surgery was 1.5 years (range 0.4–12 years), and the median body weight was 30 kg (range 15–68 kg). A total of 18 breeds were represented, including German Shepherd (6), Great Dane (4), Maremmano-Abruzzese Shepherd (4), Boxer (2), Labrador Retriever (2), Newfoundland (2), mixed breed (8), and 11 dogs of other breeds.

Complete physical examinations, CBC, and serum biochemistry investigations performed in all dogs were normal, except in one dog which had a heart murmur that was shown to be caused by mild pulmonary stenosis via echocardiography. The remote medical history of one dog reported that it had been found to be positive for giardiasis but had no symptoms, while the owner of another dog reported that her dog had had recurrent episodes of gastric dilation. All dogs were brought to the clinic for elective prophylactic gastropexy, except for one dog that had suffered from an episode of GDV that had been resolved with medical therapy.

Additional procedures were performed in 22 dogs, with 19 dogs undergoing laparoscopic ovariectomy, 2 dogs undergoing prescrotal castration, and 1 dog undergoing laparoscopic ovarian residue removal.

The total surgical time (from first skin incision to last port closure), including additional procedures, was 30 min (median, range 10–59 min). The median duration of laparoscopic gastropexy (from the insertion of the instruments in the ports to suture fulfillment) was 12 min (range 4–30 min). The median length of the suture was 3 cm (range 2–4 cm), while the median number of suture bites was 3 (range 3–4). In all procedures, only one unidirectional barbed suture line (0 Filbloc, 15 cm, PDO monofilament long term absorption, Assut Europe, Roma, Italy; HSRG 36 mm-1/2 tapper cutting, Assut Europe, Roma, Italy) was used. In two cases, a longer suture line was employed (0 Filbloc, 45 cm, PDO monofilament long term absorption, Assut Europe, Roma, Italy; HSRG 36 mm-1/2 tapper cutting, Assut Europe, Roma, Italy). No observable tearing or suture loosening was noted in subjective evaluations performed at the end of the procedure.

No major intraoperative complications occurred, and no surgery required conversion to laparotomy. Minor intraoperative complications included cutting of the suture line during the placement of the endoclip (4), needle folding (1), needle holder breakage (1), and mild self-limiting gastric serosa hemorrhage (1). All procedures were performed in a day hospital.

Immediate postoperative complications occurred in three cases, including incisional inflammation of one port (2) and incisional seroma formation (1), each of which were resolved within 2–3 days and did not require additional veterinary intervention. No complications were recorded two weeks postoperatively.

Follow-up information was available for 39 dogs, and no gastric dilation or GDV episodes were reported. The last clinical follow-up assessment was performed 9 months (median, range 3–14 months) after surgery. In three cases, owners reported one episode of vomiting more than three months postoperatively, and one dog experienced two days of acute vomiting that resolved with symptomatic therapy four months after surgery. There was mild weight loss in one dog, while two dogs gained weight. According to the owner, the dog that suffered from recurrent gastric dilation before surgery did not show any symptoms during the observation period. All clients were satisfied with the outcome of the procedure and stated that they would recommend it to other people. The quality of life of each dog on a scale of 1 to 10 was rated as 10 (median, range 8–10).

**Figure 3:**
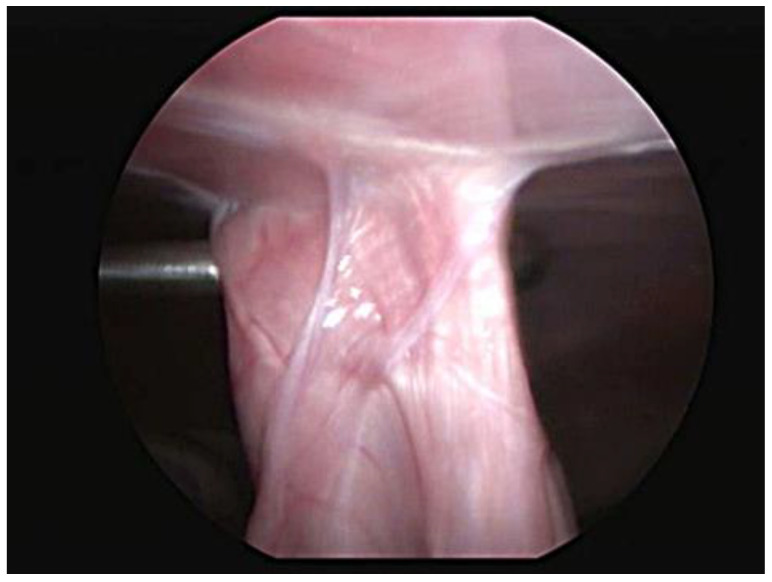
Laparoscopic view of the intact gastropexy 6 months postoperatively.

Abdominal ultrasound examinations were available for 29 cases. The last evaluation, which was performed at a median of 6 months (range 3–12 months), showed an intact adhesion at the gastropexy site in all dogs. Four dogs were examined twice, at 3 and 6 months after the total laparoscopic gastropexy, and no significant differences were noted. One dog underwent a CT scan for a complex femur fracture at 8 months postoperatively, and both the endoclip and pexy site were identified. One case had a laparoscopic vasectomy 6 months after surgery, at which time the gastropexy was proven to be intact, and adequate adhesion formation between the body wall and pyloric antrum was observed (Figure 3).

## 4. Discussion

This clinical prospective multicenter study suggests that it is possible to perform a total laparoscopic prophylactic gastropexy procedure with the use of two needle holders and one self-retaining knotless unidirectional barbed suture line without making incisions or abrasions on either the abdominal or gastric wall, creating an intact gastropexy long term. To our knowledge, this is the first time this technique has been described.

Deroy et al. [23] previously described a very similar method where the peritoneum and gastric seromuscular layer were not intentionally traumatized using an endoscopic suturing device (Endo Stitch; Medtronic, Minneapolis, MN, USA), and a median of seven bites were performed. On the contrary, in our study, two laparoscopic needle holders were always used, and the median suture length was 3 cm with a maximum of four bites. It has been shown that it is not the number of suture lines that influences the biomechanical properties of a gastropexy but their length [26]. Usually, in a total laparoscopic gastropexy procedure, only one suture is performed [22,23,24,25]. Even though it has been calculated that a 2 cm long gastropexy is weaker than a 4 cm one, the 2 cm one is supposed to be adequate to avoid a torsion. However, the tensile strength of a pexy required to prevent a GDV in vivo has not yet been determined [7,19,20,26], nor has the number of suture bites required to produce a reliable gastropexy [21].

In almost all cases, a 15 cm polydioxanone monofilament unidirectional barbed suture (Filbloc 180-210, Assut Europe, Roma, Italy) with a textile resistance of 70% at 28 days, 55% at 42 days, and complete absorption at 180–210 days was used. This type of suture is indicated for the suturing of soft tissues where long-term absorption is required and in surgeries where knot-tying is challenging and time-consuming, such as in laparoscopic surgery and robotic surgery in humans [27,28]. The unidirectional barbs engage with the tissues and keep the tension constant along the entire strand, preventing the suture from slipping. Twice, a 45 cm suture was used due to the lack of a 15 cm one, and in those cases, the surgical time lengthened due to the reduced handling.

The formation of a permanent adhesion between the seromuscular layer of the stomach and the transversus abdominis muscle occurred in the absence of any incision or abrasion of the tissues. This could be explained by the fact that the suture barbs, in association with the manipulation of the organs, generated sufficient trauma to promote fibrous adhesion. In an experimental study [29], it was shown that barbed sutures caused greater postoperative adhesion formation than traditional sutures. Unfortunately, in dogs, the minimum amount of tissue damage leading to the formation of permanent adhesions remains unknown [23].

The median total surgery time and the median gastropexy surgery time in our study were shorter than those reported in previous similar studies [22,23]. Unfortunately, it was not possible to compare the timing related to gastropexy surgery alone, because in this study, the time was calculated from the insertion of the instruments inside the ports to suture completion, while in other works, it was calculated as the difference between total surgery time and the duration of additional procedures. Nevertheless, it is thought that the reduction in surgical time is due to the fact that only one suture line is used, neither incisions nor abrasions are made on the abdominal wall and the stomach, and the terminal button of the Filbloc makes it possible to proceed with the suture without prior anchoring. Additionally, in two studies [8,21], surgical times were reduced when surgeons used two laparoscopic needle drivers instead of an endoscopic suturing device.

The main intraoperative complications were technical problems (suture line cut during endoclip placement, needle folding, needle holder breakage), and these resulted in the lengthening of surgical times without interfering with the final outcome of the procedure. Gastric serosa hemorrhages that occurred were mild and did not require any intervention. Pneumothorax [25], suture breakage [23], subcutaneous emphysema at port sites [12,24], or splenic lacerations [8,22] did not occur.

The dogs were monitored on the day of the surgery, and none presented vomiting, postoperative regurgitation, or lethargy [8,23]. The incidence of immediate postoperative complications, such as wound-related problems (bruising, swelling, or erythema of the incision sites) was comparable to those reported for total laparoscopic gastropexies [12,22,23,24], and these issues were resolved within 2–3 days without veterinary intervention.

During postsurgical monitoring, there were no episodes of GDV, but a longer period of follow-up monitoring should be performed to verify the efficacy of the gastropexy procedure [16]. Several studies have tried to demonstrate the benefits of gastropexy in breeds considered to be predisposed to episodes of GDV; however, the individual lifetime risk of a dog for the development of GDV is still unknown [1,6]. Gastropexy is still a controversial topic for both veterinarians and owners; it is known to avoid gastric torsion in all cases, but the risk of gastric dilation and its serious consequences still remains [30]. One owner reported that, since undergoing gastropexy, their dog had no longer had swelling of the abdominal wall or frequent belching. Analyzing the anamnesis of this case and observing the preoperative radiograph, which showed partial dilation of the stomach, it was thought that it could have been a patient who suffered from recurrent dilations. In a case report, the resolution of recurrent gastric dilation with a left-side gastropexy was described [31]. Four dogs presented episodes of vomiting over 3 months postoperatively, including one dog that required medical therapy. These were all self-limiting and isolated episodes, and the owners blamed them on the ingestion of something inappropriate by the dog. The loss of weight by one dog was explained by the owner to be a consequence of a hypocaloric diet.

Ultrasound evaluation showed an intact gastropexy in all dogs examined. The pyloric antrum of the stomach was observed to be in contact with the right abdominal wall, and no slide sign of the viscera was noted at the site of the pexy. The titanium endoclip was also visible, and it was surrounded by a thin avascular capsule. The examined reports were very similar to those previously described [2,3,5,12,22,23,24,32]. Ultrasound is a noninvasive, repeatable method, and it usually does not require sedation of the animal. However, it cannot be used to establish the quality or strength of a gastropexy. A postoperative laparoscopic evaluation is more useful for assessing the strength of the adhesion [16]. However, due to the invasiveness of the procedure, it was not included in the postoperative follow-up examinations unless another laparoscopic procedure had to be performed. In one case, it was possible to observe the gastropexy site during laparoscopic vasectomy. For this, the stomach was pulled away from the body wall with Babcock forceps to subjectively verify the strength of the adherence, and it was evaluated as being adequate. Computed tomography (CT) can also be considered a valid method to assess the position of the stomach following a gastropexy procedure, but it is much more invasive and expensive [33].

This study had several limitations. A control group of dogs that have undergone an incisional total laparoscopic gastropexy should be used in the future to assess whether the differences in time and effectiveness are significant or not. Biomechanical testing could not be performed in this study due to the nature of the research. Similarly, laparoscopic checks and biopsies were not used because they were considered too invasive. Therefore, a permanent gastropexy was confirmed only via ultrasound examinations. Limitations in addition to those already discussed included the small sample size and lack of a normal distribution.

## 5. Conclusions

The results of this study suggest that total laparoscopic gastropexy can be performed with a single self-retaining knotless unidirectional barbed suture and two needle holders without performing incisions or abrasions on either the gastric seromuscular layer or the transversus abdominis muscle. We conclude that this technique is safe, fast, and less technically-demanding when compared with other total laparoscopic gastropexies, and it has a low morbidity rate when performed by surgeons who are skilled in carrying out minimally invasive procedures. It appears to be a suitable alternative to other methods. Longer-term clinical evaluations, a larger sample size, biomechanical tests, and biopsies are recommended to further assess the efficacy and benefits of this type of surgery.

## Data Availability

No new data were created or analyzed in this study. Data sharing is not applicable to this article.
